# Comparative genome analysis: selection pressure on the *Borrelia vls *cassettes is essential for infectivity

**DOI:** 10.1186/1471-2164-7-211

**Published:** 2006-08-16

**Authors:** Gernot Glöckner, Ulrike Schulte-Spechtel, Markus Schilhabel, Marius Felder, Jürgen Sühnel, Bettina Wilske, Matthias Platzer

**Affiliations:** 1 Genome Analysis Group, Leibniz Institute for Age Research – Fritz Lipmann Institute, Beutenbergstr. 11, 07745 Jena, Germany; 2 Max-von-Pettenkofer Institut für Medizinische Mikrobiologie und Hygiene München, Germany; 3 Biocomputing Group, Leibniz Institute for Age Research – Fritz Lipmann Institute, Beutenbergstr. 11, 07745 Jena, Germany

## Abstract

**Background:**

At least three species of *Borrelia burgdorferi *sensu lato (Bbsl) cause tick-borne Lyme disease. Previous work including the genome analysis of *B. burgdorferi *B31 and *B. garinii *PBi suggested a highly variable plasmid part. The frequent occurrence of duplicated sequence stretches, the observed plasmid redundancy, as well as the mainly unknown function and variability of plasmid encoded genes rendered the relationships between plasmids within and between species largely unresolvable.

**Results:**

To gain further insight into *Borreliae *genome properties we completed the plasmid sequences of *B. garinii *PBi, added the genome of a further species, *B. afzelii *PKo, to our analysis, and compared for both species the genomes of pathogenic and apathogenic strains.

The core of all Bbsl genomes consists of the chromosome and two plasmids collinear between all species. We also found additional groups of plasmids, which share large parts of their sequences. This makes it very likely that these plasmids are relatively stable and share common ancestors before the diversification of *Borrelia *species.

The analysis of the differences between *B. garinii *PBi and *B. afzelii *PKo genomes of low and high passages revealed that the loss of infectivity is accompanied in both species by a loss of similar genetic material. Whereas *B. garinii *PBi suffered only from the break-off of a plasmid end, *B. afzelii *PKo lost more material, probably an entire plasmid. In both cases the *vls *gene locus encoding for variable surface proteins is affected.

**Conclusion:**

The complete genome sequences of a *B. garinii *and a *B. afzelii *strain facilitate further comparative studies within the genus Borrellia. Our study shows that loss of infectivity can be traced back to only one single event in *B. garinii *PBi: the loss of the *vls *cassettes possibly due to error prone gene conversion. Similar albeit extended losses in *B. afzelii *PKo support the hypothesis that infectivity of *Borrelia *species depends heavily on the evasion from the host response.

## Background

Infections with *Borrelia *species cause thousands of human disease cases per year [1]. The major causative agents of this disease are three species of the *Borrelia burgdorferi *sensu lato complex (Bbsl), *B. burgdorferi *sensu stricto, *B. garinii*, and *B. afzelii *[2-4]. As was shown previously using phylogenetic tree reconstructions [5,6]*B. garinii *and *B. afzelii *are more closely related than either of them to *B. burgdorferi *sensu strictu, which branches at the basis of the three-species tree. *Borreliae *are obligatorily bound to host organisms for survival. During their life cycle they switch from the invertebrate host tick (Ixodes spec.) to various vertebrate hosts via a tick bite. The range of vertebrates and invertebrates used as hosts is thought to be mediated by factors encoded mainly on a large number of different plasmids found in *Borreliae *genomes. A further characteristic of the plasmids is their wealth of paralogous genes. During passage plasmids can be lost due to the lack of selection pressure as was shown recently [7]. This loss can be accompanied by the inability of *Borreliae *to prosper in the host. Furthermore, it is thought that *Borreliae *plasmids are not stable and are frequently rearranged leading to differing plasmid content within a species [8,9]. To some extent the chromosome is also involved in fission/fusion events. It was shown that the right end of the chromosome of *B. burgdorferi *strains is variable due to its ability to catch plasmid material [10]. The high variability of *Borreliae *genomes has so far hindered a concise description of the genome properties of *Borreliae *based on plasmid size estimates and hybridizations alone.

Comparative genomics aims at the description of related organisms based on their common and discernible genetic material [11-13]. Furthermore, it enables the evaluation of the relationship between genotype and phenotype, if clear-cut phenotypic differences are described for the species in question. In case of prokaryotes a genome comparison can e.g. discern common genomic backbones from otherwise acquired genetic material [14].

To date the complete genome of *B. burgdorferi *sensu stricto (B31) is known [15]. It exhibits a wealth of circular as well as linear plasmids, some of which are nearly identical [8]. In a more recent study we reported on the genome of a *B. garinii *strain [16]. This comparative genomics approach showed clearly that three genomic elements, the chromosome and plasmids cp26 and lp54, are common to both *Borrelia *species and, more strikingly, collinear. At the same time we were able to show that some plasmids are confined to *B. burgdorferi *sensu stricto, since no trace of these plasmids including their coding capacity could be found in the whole genome shotgun sequencing (WGSS) data of *B. garinii *PBi. Yet, at this time it was not possible to give a clear picture of the plasmid content of *B. garinii *PBi, since plasmidal sequences were distributed over 36 sequence contigs that did not represent entire plasmids.

In this study we determine the complete sequences of the *B. garinii *PBi plasmids and the whole genomic sequence of a third species, *B. afzelii *PKo [GenBank: CP000397, GenBank: CP000398, GenBank: CP000399, GenBank: CP000400, GenBank: CP000401, GenBank: CP000402, GenBank: CP000403, GenBank: CP000404, GenBank: CP000405, GenBank: CP000406]. Furthermore, we wanted to shed light onto the requirements for pathogenicity in these species. For this purpose, sequences from pathogenic low passage and from high passage strains, which have lost their pathogenicity, were combined to yield the entire genome sequences of these two *Borrelia *strains.

## Results

### Chromosome and plasmid sequences

As we had previously shown by the genome analysis of an early (12^th^) passage of *B. garinii *PBi [16] a coverage as low as 3× is sufficient as a basis for genome analysis of Bbsl species. Extending this approach we applied low coverage WGSS to a late (300^th^) passage of *B. garinii *PBi as well as to early (8^th^) and late (70^th^) passages of *B. afzelii *PKo. The resulting sequences were assembled species-wise to give a coverage of around 6 for each genome (Table 1). Of the *B. garinii *genome we sequenced equal amounts of clones from high and low passage strains; of the *B. afzelii *genome only one third of all sequences were derived from the low passage strain. Employing additional gap closure procedures, we obtained nearly gap free genomic sequences. Contigs or parts thereof consisting only of low passage DNA derived clones were considered to have been lost during cultivation.

### Collinearity and indels of the Bbsl chromosomes

**Figure 1:**
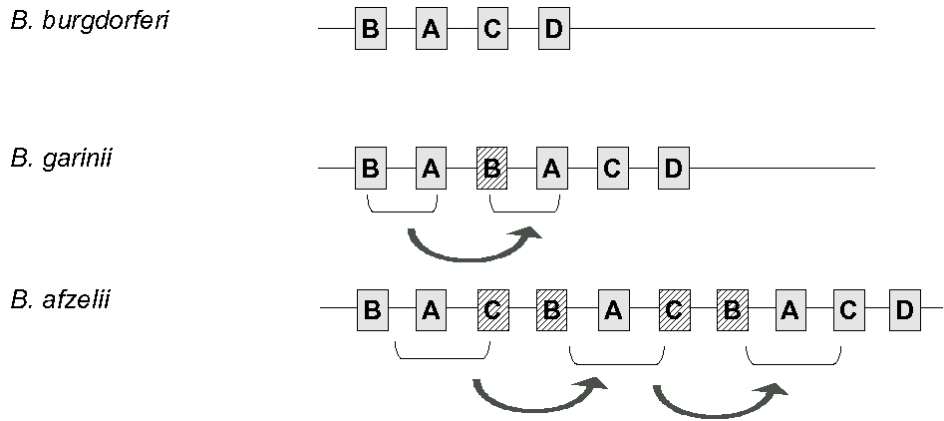
Chromosomal duplications of the bmp cluster genes in different *Borrelia *species. Grey boxes are functional genes, hatched boxes denote pseudogenes. Arrows indicate duplication events.

Both *B. garinii *PBi and *B. afzelii *PKo genomes have collinear chromosomes of comparable sizes (Table 1) with only minor insertions and deletions. Pairwise alignments using stretcher revealed that most indel positions in *B. garinii *PBi and *B. afzelii *PKo are shared compared to *B. burgdorferi *B31 (Table 2) indicating a closer relationship between these two species than to *B. burgdorferi *B31. Most indels are small, but we also observed three regions exceeding 500 bp in *B. afzelii*. One of these differences between the chromosomes of Bbsl species is attributable to differing sequences at the origin of replication in the middle of the chromosome. The second affects the *bmp *locus at position 394,080 of the *B. burgdorferi *chromosome. We previously noticed that this locus is incompletely duplicated in *B. garinii *PBi compared to *B. burgdorferi *B31 [16]. Now, with a third sequence at hand we see that this locus seems to be instable in all species. In *B. afzelii *PKo this locus is incompletely triplicated. Most interestingly, the duplication events observed produce only functional copies of *bmpA*. All other duplicated *bmp *genes are rendered truncated during the duplication event (Fig 1). The third large indel affects a further outer membrane protein gene (*lmp1*), thus contributing to the diversity of the protein orthologs and possibly to the variations in the host pathogen interaction.

### Striking differences in the plasmid content

The Bbsl plasmid fraction varies significantly between different species. Whereas the *B. garinii *PBi plasmids comprise only 29 % of the genome, the *B. afzelii *PKo plasmids add up to 36 % (Table 1). Yet, this is still well below the plasmid content of *B. burgdorferii *sensu stricto (47 %) [8]. Assemblies of the respective reads yielded 11 plasmids for the *B. garinii *PBi genome and at least 16 for the *B. afzelii *PKo genome (Table 3). Circular plasmids can be defined as finished once no gap remains in the sequence and the ring is closed. Using this criterion, we were able to define three circular plasmids in *B. garinii *PBi. In *B. afzelii *PKo we detected two circular plasmid types. On of these types comprises a group of eight very similar plasmids resembling the situation in *B. burgdorferi *B31. We were not able to completely resolve this group of eight plasmids since they are highly similar, if not identical, over long sequence stretches. This renders impossible the correct assignment of a considerable number of clones to a specific plasmid and PCR approaches to close gaps between plasmid segments. Only one plasmid with a size of over 30 kb could be entirely finished. Yet, the extraordinarily high identity of the group members to each other led us to the conclusion that all members of this group are circular.

We tested using PCR approaches, whether any of the remaining sequence contigs could be only part of another plasmid. Using this method, we closed 15 gaps between segments with unambiguous sequences. The remaining segments could not be linked together, since no or no unique PCR products could be produced with any of the combinations of ends possible. We sequenced several of the PCR products from primer combinations, which gave rise to more than one PCR product. Sequences derived from all these products were either of low sequence quality indicating PCR product mixtures or located elsewhere in the genome. Thus, since we were not able to find additional links between the sequence segments described here, we conclude that most of the described segments are indeed individual plasmids of these species and are supposed to be linear. The plasmid size range extends from 6 to over 60 kb (Table 3). Plasmid names were chosen according to size and to their circular (cp) or linear (lp) nature. The sequence reads of the small fragments in the range of 6 to 15 kb in both species were only found in low passage strains (see below).

The strain of *B. garinii *we present here has the fewest number of plasmids of all *Borrelia *species analysed so far. Including the indispensable cp26 and lp54 orthologs, there are three circular and eight linear plasmids. Conversely, the *B. afzelii *PKo genome comprises nine circular and seven linear plasmids.

### Plasmid homologies between *Borrelia *species

We wondered whether we would be able to detect additional relationships between plasmids of different species besides the clearly homologous plasmids cp26 and lp54. In a first step we examined the relationships within a species. Most prominent in this respect are the *B. burgdorferi *B31 circular plasmids in the range of 30 kb, which are highly similar to each other with only minor sequence differences over large parts of the plasmids. We observed a homologous group of plasmids also in *B. afzelii *PKo, where we found 8 circular plasmids with a size of 30 kb. This group may even include further plasmids, since we were not able to assemble this group fully. Furthermore, despite the length differences between the two circular plasmids cp29 and cp31 the *B. garinii *PBi plasmids constitute a similarity group as shown by tupleplot analysis [see Additional file 1]. A three-species comparison revealed that all these circular plasmids belong to the same group and originated from a common ancestor. Most strikingly and despite their high sequence diversity, which renders an interspecies alignment difficult, these plasmids exhibit a conserved collinearity [see Additional file 1]. Intriguingly, these plasmids are amplified and diversified (i.e. they exhibit polymorphisms to each other) in all species albeit with differing copy numbers. The most remarkable distinguishing feature within this group of plasmids is the length difference observed in *B. garinii *PBi. An all-to-all comparison of the other plasmids showed that there are additional plasmid groups with a common ancestor although with slightly different lengths and minor unique sequence portions (Table 4 and Additional file 1).

To classify these plasmids we had to relax the criteria, according to which plasmids are grouped together. Thus, the minimal requirement for a group is to have detectable similarities (using tupleplot, see materials and methods) over a significant part of their sequence length irrespective of rearrangements. As is listed in Table 4 we can define six different groups according to their common presence in all Bbsl species. Group VI is the least well defined group since the similar sequence stretches are divided up in *B. burgdorferi *between three different plasmids. All other groups contain plasmids, of which the similar DNA sequences seem to be stably inherited within individual plasmids.

The relatedness between the plasmid content of *B. afzelii *PKo and *B. garinii *PBi is notable. Their plasmids within the same groups are more related to each other concerning size, shared similarity and extent of rearrangements than to the *B. burgdorferi *sensu stricto counterparts. Conversely, the otherwise highly amplified group III plasmids have undergone a reduction in the *B. garinii *PBi genome, whereas some other group specific plasmids were amplified (groups IV and VI).

### Low passage versus high passage strains – genomic differences

As mentioned above we did not use a uniformly grown culture for the construction of whole genome shotgun libraries. Instead, we used low and high passage strains of the same isolate. The major difference between low and high passage is the loss of infectivity during repeated passages [9,17,18]. These strains should have accumulated only minor numbers of single base differences due to the short time of separation. Indeed, single nucleotide exchanges on the chromosomes were very rare. We found only 26 such events in *B. garinii *PBi and even less in *B. afzelii *PKo (4). We think that loss of infectivity is accompanied by larger changes in the genome of the strains, i.e. loss of whole parts of the genetic material.

**Figure 2:**
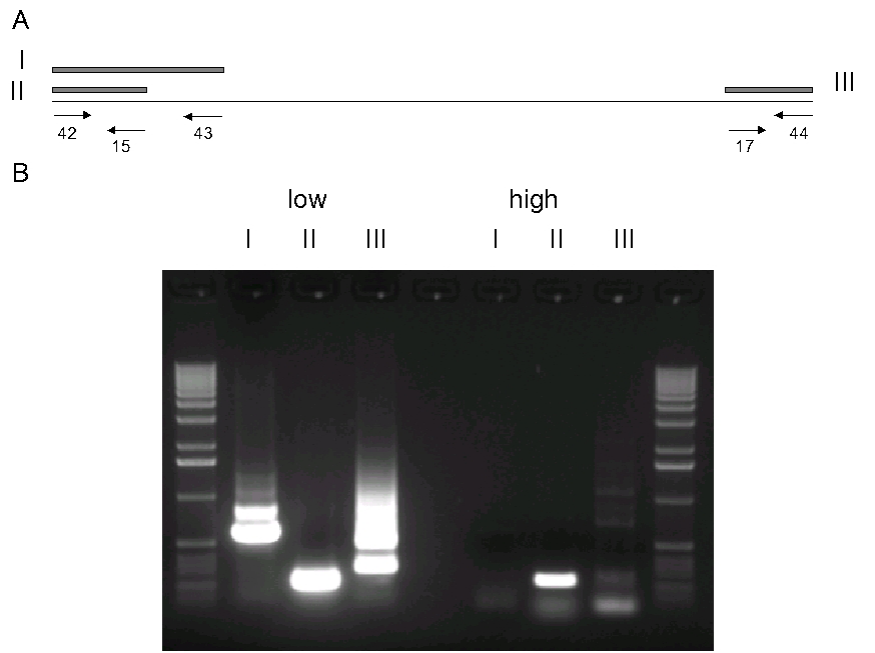
The *vls *cassette fragment in low and high passage strains of *B. garinii*. A: Designed primers and expected PCR products from the 6 kb low passage DNA fragment containing *vls *cassettes of *B. garinii*. B: Electrophoresis of PCR products obtained with various primer combinations. Roman letters indicate primer combinations from A. Low and high refer to DNA from low and high passage strains, respectively.

In case of *B. garinii *PBi we found all plasmids represented in both the low passage as well as the high passage culture. This is in good agreement with previous findings, where no plasmid differences could be detected in low and high passage strains of *B. garinii *PBi [9]. Yet, a small fragment of 6 kb was not detectable in the high passage strain shotgun data. An analysis of the gene content revealed, that nearly all of the 6 kb segment is occupied by cassettes of the *vls *gene cluster. This cluster was previously mapped to a 28 kb linear plasmid in *B. garinii *[19]. Yet, this fragment could not be assigned to one of the other plasmids despite testing all combinations possible using specific primers for all linear plasmid ends. It is very unlikely that we missed an entire plasmid with our shotgun cloning approach. Thus, this failure may be due to the repetitive nature of the *vls *locus and/or its frequent cassette switchings [20,21], which makes the generation of matching unique primers impossible. Using different primers on this fragment we were able to amplify several DNA species of different lengths from the low passage strain (Fig. 2). Amplification in the high passage strain yielded a product only for the leftmost primer pair. All PCR products were sequenced and were confirmed to be derived from the correct genome position. This indicates that most of the *vls *cassettes and the *vls *gene itself was lost during passaging. A Southern blot analysis confirmed that the entire vls locus is missing in the high passage strain [see Additional file 1].

The genomic losses of *B. afzelii *PKo are more severe than those of *B. garinii *PBi. In *B. afzelii*, three segments with the cumulated size of 30 kb seem to be missing in the high passage strain. Previous results suggested the loss of a 31 kb and a 6 kb plasmid from the high passage strain of *B. afzelii *PKo [9]. Despite our inability to connect these segments via PCR it is very likely that these segments constitute two linear plasmids in *B. afzelii *PKo, The assembly of the plasmids consists almost entirely of low passage strain DNA material. Sequence reads of two clones derived from the high passage strain suggest that this loss is not achieved as a sudden event but progresses gradually within the culture. One of the segments (8 kb) contains a part of the *vls *gene cluster. Moreover, we found additional reads with similarities to *vls *gene and cassette fragments solely in the low passage shotgun data (adding up to 4 kb). Again, we were not able to connect all these reads to other plasmid contigs using PCR approaches. We therefore conclude that the *vls *cluster is missing from the high passage strain of *B. afzelii *PKo. Previous Pulsed Field Gel experiments indicated the loss of a 6 kb and a 31 kb linear plasmid from the high passage strain. Thus, the 8 kb and the 15 kb fragment together with the vls cluster should form this 31 kb linear plasmid, the 6 kb fragment is therefore one entire linear plasmid. Interestingly, the fragments of *vls *genes we sequenced do not properly align with the previously published sequence of the entire 8 kb *vls *locus of *B. afzelii *[21]. This finding demonstrates that this locus is highly polymorphic due to casette switching and homologous recombination.

## Discussion

### *Borrelia burgdorferi *sensu lato chromosome differences

The chromosomes of Bbsl species harbour only minor differences between them. This includes right arm extensions, which may be derived from linear plasmids and three major indels. Interestingly, two of these indels affect outer membrane proteins, thus contributing to the surface diversity between the species. This is most clearly seen at the *bmp *locus, where the copy number varies and the duplicated genes deviate slightly from the original sequence (Fig. 1). Since these differences are fixed in the respective genomes, it is likely that this diversity may contribute to subtle variations in the host range and initial success to invade the host.

### Homologous plasmid groups in *Borrelia *species

The analysis of the *B. burgdorferi *plasmids [8] showed a wealth of similar regions of differing sizes residing on several plasmids. Our previous genome analysis of *B. garinii *PBi resulted in the assembly of the plasmid compartment in 37 segments, which in comparison to the *B. burgdorferi *plasmids revealed both common and unique sequences [16]. Yet, due the fragmented data set it was impossible to directly compare the entire plasmids. This work now presents the nearly gap-free sequence of the plasmids not only from *B. garinii *PBi but also from *B. afzelii *PKo.

All species contain three seemingly invariable and indispensable parts, the chromosome, a cp26 like circular plasmid, and an lp54 like linear plasmid. They are relatively well conserved and collinear. All other plasmids cannot directly be aligned between species due to a low sequence similarity. Furthermore, comparisons of genes on amino acid level can obscure real relationships between plasmids due to unclear orthology. Yet, tupleplot analysis revealed a striking overall similarity of long stretches of some plasmids with others within and between species. Therefore, despite high sequence and length variability, as well as broken collinearity, we can describe groups of plasmids apparently derived from a common ancestor (Table 4). The most prominent group consists of circular plasmids in the size range of 30 kb, which are highly amplified in *B. burgdorferi *B31 and *B. afzelii *PKo but not *B. garinii *PBi. The two *B. garinii *counterparts to this group are more diverse in size and sequence than in the other species indicating an accelerated evolution towards a higher diversification and possible secondary reduction of this group. The plasmids of this group encode outer membrane factors, erp proteins, which are thought to be involved in host range definition [22]. Since *B. garinii *PBi therefore is able to produce only two different erp proteins, the host range of this species is potentially reduced compared to the other two species. Groups IV and V share large parts of their sequences between species albeit with some intra-plasmid rearrangements. The weakest homology shows group VI where similar plasmid segments are dispersed over different plasmids in *B. burgdorferi *B31. Intriguingly, intra-plasmid divergence and shuffling seems to be favoured over inter- plasmid rearrangements. This finding indicates that the *Borrelia *genome is more stable and less prone to recombinations than previously thought [8]. Unique plasmids (i.e. with less than 50 % sequence similarity to any other plasmid) occur in all three species. These may contribute to the disease variations or adaptation to different environments. A previous study relying on Southern blots using probes derived from *B. burgdorferi *B31 plasmids showed relationships between the probes and plasmids of different strains and species [23]. Only one probe was used per plasmid restricting conclusions on homologies between plasmids to only the probe specific regions. Yet, in most cases this study is in agreement with our findings if possible cross-hybridisations with small similar regions are taken into account. The most prominent difference is within group VI. Our results indicate homology of lp25 to lp21-2 and lp22, whereas Palmer et al. detected homology to a 30 kb linear plasmid. We therefore conclude that the 6 kb fragment harbouring th vls gene cluster is most likely connected to one of these plasmids. This is also in good agreement to previous findings, where the vls cluster of *B. garinii *was located on a 28 kb linear plasmid [19].

From all sequence information, we can define three evolutionary trends: i) in *B. garinii *some plasmids (group IV and VI) were duplicated and diversified at the expense of plasmids from group III that are highly amplified in other species ii) unique plasmids in each species were either acquired by horizontal transfer [24] or diversification of existing plasmids. iii) plasmids of *B. afzelii *PKo and *B. garinii *PBi are more similar to each other than to those of *B. burgdorferi *B31 as it is especially pronounced in group VI. This is in good agreement with phylogenetic tree reconstructions using different genes [5,6] and implies that at least these defined plasmid groups evolved without horizontal gene transfer.

Together, the three way comparison presented here provides a concise description of the plasmids of Bbsl and offers new insights into their evolutionary origin.

### Loss of genetic material and infectivity during passage

*Borrelia *species can lose their infectivity, when passaged several times without selection pressure for infectivity. This loss is accompanied by loss of genetic material [18,25]. It is not possible to estimate the extent of this loss based solely on PFGE or PCR, especially if the whole genomic sequence is not known. We therefore sequenced and analysed low and high passage strains of *B. garinii *as well as of *B. afzelii *together to be able to describe all sequence differences and to determine the cause of lost pathogenicity.

Surprisingly, not a whole plasmid but only a part of a plasmid was lost during passage in *B. garinii *PBi. The remaining part of the plasmid was either retained by chance or is essential. The lost part consists of *vls *gene cassettes whose frequent exchange is involved in the escape of *B. burgdorferi *from the host response [19,20]. Thus, this selective loss may be caused by the failure to correctly repair the locus after recombination. Absence of selection pressure then allows this clone with the truncated plasmid to prosper in the culture. In order for the entire culture to lose the locus, cassette switching errors must be frequent or the mutated clone has a substantial growth advantage.

In *B. afzelii *PKo an entire plasmid is missing in the high passage strain. This plasmid also contained the *vls *genes as could be concluded from the presence of a few *vls *cassette derived reads. Loss of entire plasmids could occur if the plasmids are not correctly divided up between the daughter cells during division. Yet, we found that two sequences in the assembly of the fragments (Table 3) are derived from the high passage strain DNA indicating a two step process of plasmid loss. In this scenario the *vls *cluster is lost first as in *B. garinii *PBi, and in a second step the non essential remainder of the plasmid is lost successively.

Interestingly, in the tick vector the vls locus seems to be silent [26] and thus is stable in this host. This can explain, why the accidental loss does not occur in nature.

Thus, loss of infectivity is accompanied in both species by the loss of the *vls *locus. While this loss seems to be the sole reason for the loss of infectivity in *B. garinii *PBi, the influence of the additional material lost in *B. afzelii *PKo on infectivity remains to be determined. Yet, we believe that this additional loss occurred only by coincidence due to the non essential nature of the remaining parts of the plasmid. This leaves the *vls *locus as the prominent but fragile cause for the successful thriving of *Borrelia *species in their respective vertebrate hosts.

## Conclusion

Comparative sequencing is a valuable tool to discern species specific and common genetic material. Our analysis of genomes from *B. garinii *and *B. afzelii *strains together with the previously described *B. burgdorferi *B31 genome shows that more plasmids are indispensable for *B. burgdorferi *sensu lato than previously thought. In *Borreliae *lack of selection can lead to the loss of genetic material accompanied by a phenotype change. A base by base analysis of these losses as we made it here can not only reveal genes important for the phenotype change upon their loss but also gives clues about underlying mechanisms for the losses. Further studies on additional low and high passage strains should reveal whether the loss of the vls cassette is the major or even single causative event which leads to loss of infectivity.

## Methods

The *B. garinii *strain PBi (*OspA *serotype 4) is a CSF isolate from a German patient with neuroborreliosis. Passage 12 is still infectious for gerbils, but infectivity was lost between passage 30 and 60 [27]. *B. afzelii *strain PKo was originally isolated from human skin [28], the early passage is still infectious as can be demonstrated with gerbils. Early (8^th ^subculture) and late (70^th ^subculture) passages of *B. afzelii *PKo and a late passage of *B. garinii *PBi (300^th^) were grown in MVP-medium as described [29].

DNA was extracted using the Genomic DNA Bufferset (Quiagen GmbH, Hilden) and Genomic tip 500/6 and 100/6 (Quiagen, GmbH, Hilden). Genomic libraries with a target insert size of 1.5 kb using total DNA were constructed as described previously [16]. From all libraries sufficient clones were sequenced from both ends to give a sequence coverage of at least 5× per species (see Table 1).

All sequences were checked for similarity to chromosomal or cp26 and lp54 sequences of *B. burgdorferi *using BLAST [30] and assembled separately as described previously [16]. All other plasmid sequences were assembled together and manually checked for assembly errors. In the case of the multiple copies of cp30 in *B. afzelii *we used polymorphism and read pair information to generate unambiguously assembled contigs. This enabled us to define at least 8 different plasmids according to the placement of the contigs on a common backbone. Since we did not know, which contigs related to linear plasmids form a natural unit, we performed a PCR based analysis of possible connections between all segments. We tested all possible combinations of segment ends to determine combinations that produced PCR products when using whole genomic DNA as a template. Resulting PCR products were sequenced and used to close gaps in the plasmid assemblies. No further unambiguous PCR products were obtained linking the ends of the contigs. Thus, the obtained sequences represent either a full set of linear plasmids or, as may be the case for the smaller segments, missing links due to the inability to design unique primer pairs.

Initial gene finding was performed using GeneMarkS [31,32]. Annotation was done using the GenColors system [33].

The alignments between the *Borreliae *collinear chromosomes [see Additional file 1] were generated using the program stretcher, which is part of the EMBOSS package [34]. The similarity plot program tupleplot [35] with default settings was used for a systematic comparison of plasmids of all species to each other.

All data are available from the Spirochetes Genome Browser [36] and were submitted to GenBank, as well as to NCBI Trace and Assembly Archives.

## Authors' contributions

GG, BW, and MP conceived the study, US-S and BW provided material and input on the manuscript, MS, MF, and JS performed the bioinformatics. GG wrote the manuscript. All authors read and approved the final manuscript.

## Supplementary Material

Additional File 1 Analysis of plasmid relationships and losses. The additional file contains tuple plot analysis for all plasmids named in the text and a Southern analysis of the *B. garinii *PBi low and high passage genome using the vls cassette as a probe. Click here for file

## Figures and Tables

**Table 1:** Sequence reads produced from species *B. afzelii *PKo and *B. garinii *PBi and fractions of reads from different passages

	**#**	**Length**	**Sequence reads**			**Fraction**
				
		kb	all	low passage	high passage	low passage
***B. garinii***						
chromosome	1	905	9,968	4,803*	5,165	48%
plasmid	11	372	5,596	2,587*	3,009	46%
**sum**	**12**	**1,277**	**15,564**	**7,390**	**8,174**	

***B. afzelii***						
chromosome	1	907	7,094	1,941	5,153	27%
plasmid	16	507	3,286	1,136	2,150	35%
**sum**	**17**	**1,414**	**10,380**	**3,077**	**7,303**	

*sequences from previous work [16]

**Table 2:** Indel positions in the chromosomes of *B. garinii *PBi and *B. afzelii *PKo in respect to the *B. burgdorferi *B31 genome. Only indels larger than 40 bases are listed. Positions reflect start sites on the *B. burgdorferi *genome. Asterisks denote indels where adjacent parts were combined for clarity. Highly variable regions are bold. Please note that no ancestral state can be inferred from the table.

Insertion or deletion in respect to the *B. burgdorferi *B31 genome	*B. afzelii*		*B. garinii*		Features in affected region
	length	start	length	start	

deletion	110	137904	390	138010	
deletion	160	213176	183	213184	lmp1
**deletion**	168	213392	656	213578*	lmp1
deletion	109	228785			
deletion			150	248210	
insertion	41	318180	44	318180	
**insertion**	3400	394080	1900	393751	bmp
**insertion**	2500	443408*	2500	443413*	ori
insertion	46	476276	55	476260	
deletion	183	492057	55	492057	
deletion	124	493983			
deletion	114	557027	181	556952	
insertion			200	782940	
deletion	87	788148			
deletion	144	792943	140	792945	
insertion	330	839080			
deletion	332	844698			
insertion			40	870038	

**Table 3:** Plasmid species and denominations in *B. garinii *PBi and *B. afzelii *PKo. Completely collinear and essential plasmids are in bold letters.

species	circular plasmids	linear plasmids	putative fragments
*B. garinii*	**cp28**; cp29; cp31	lp21-1; lp21-2; lp22; lp24; lp28; lp50; lp54; **lp59**	6 kb
*B. afzelii*	**cp27**; 8x cp30	lp25; lp28; lp32; lp34; **lp60-1**; lp60-2	6 kb; 8 kb; 15 kb

**Table 4:** Homologies between plasmids of *B. burgdorferi *sensu lato species.

Orthologous groups	*B. garinii*		*B. afzelii*		*B. burgdorferi*		Bbsl	
		intra		intra		intra	inter BG-BA	inter BB-BG

	plasmid	shared	plasmid	shared	plasmid	shared		

I	cp28		cp27		cp26		collinear	collinear
II	lp59		lp60-1		lp54		collinear	collinear
III	cp29; cp31; lp50	>26 kb	cp30 group	>26 kb	cp32 group	>26 kb	>26 kb	>26 kb
IV	lp28; lp21-1	17 kb	lp25		lp28-2		17 kb; collinear	17 kb
V	lp24		lp28		lp17		rearranged/80%	65 % in three segments
VI	lp21-2; lp22	14 kb	lp60-2		lp25; lp28-3; lp36	nd	contained in lp60-2; lp22 collinear over entire length	All share middle 35 %; lp36 additional 20 %

Only plasmids with significant parts of similarity are listed. Sizes of cumulated similar DNA stretches are given in kb. nd = not defined.
