# Supplementary material for: Comparative genome analysis: selection pressure on the Borrelia vls cassettes is essential for infectivity
Source: BMC Genomics. 2006 Aug 16;7:211. doi: 10.1186/1471-2164-7-211 (PMC1559707; doi:10.1186/1471-2164-7-211)

Supplemental Material. Figures 1 –3 Tuple plots for the different plasmid groups. Dots represent identical tuples ('words') in the compared sequences. Circular plasmids can have differing starting points. Figure 4 shows PFGE and Southern Blot of *B. garinii* low and high passage.

Supplemental Figure 1: Tuple plot comparison of cp29 and cp31 of B. garinii


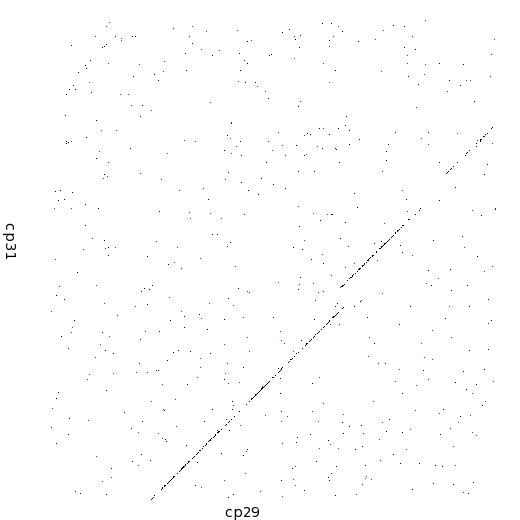


Supplemental Figure 2: Inter-species comparisons of group III plasmids. A: Comparison of B. garinii cp29 and B. afzelii cp30. B: Comparison of B. garinii cp29 and B. burgdorferi cp31.


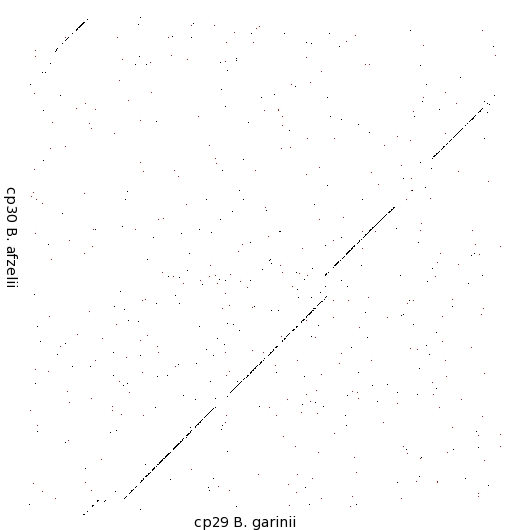
A


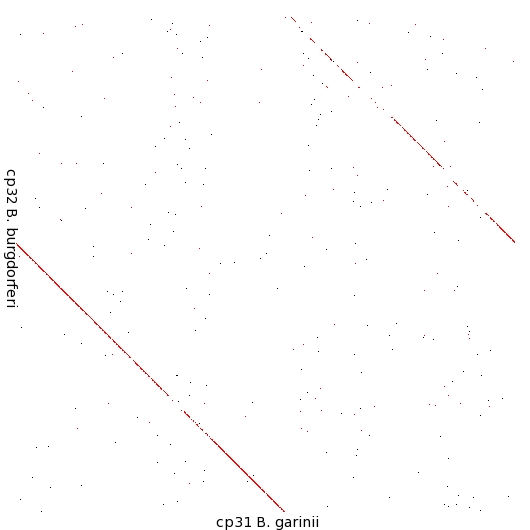
B

Supplemental Figure 3: tuple plots of B. garinii plasmids compared to B. afzelii plasmids.

A: group I, B: group II, C: group IV, D: group V, E: group VI.

A


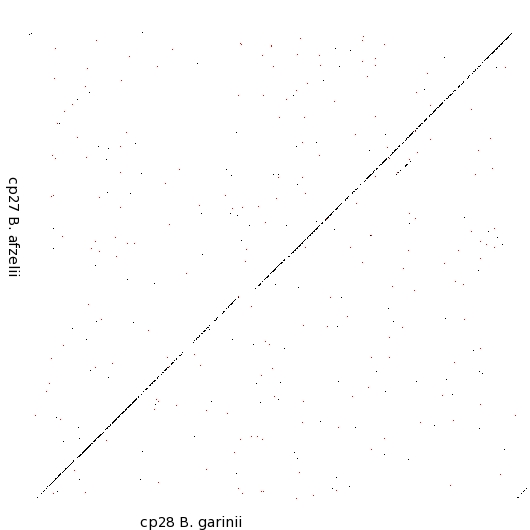


B


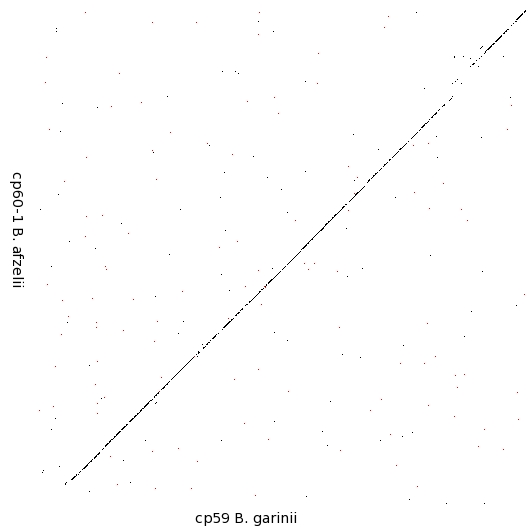


C


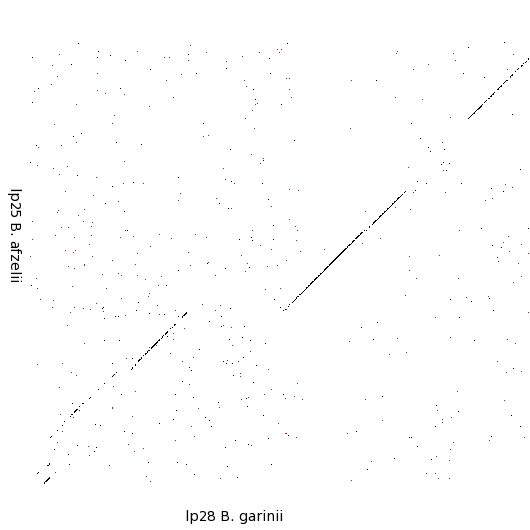


D


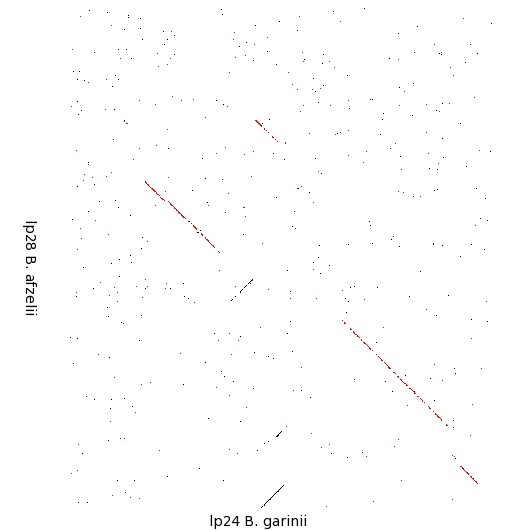


E


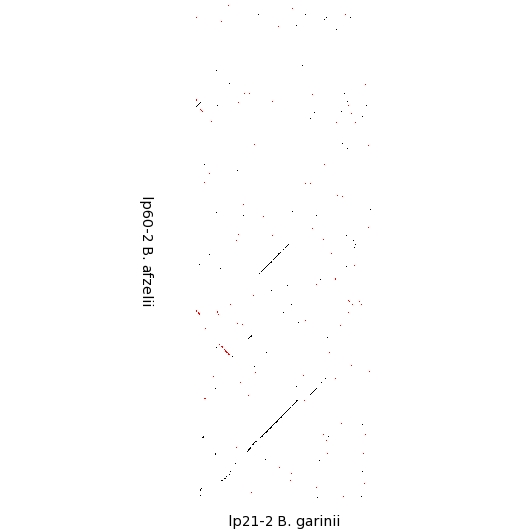


Supplemental Figure 4: PFGE (a) and Southern Blot (b) of *B. garinii* low and high passage strains. The probe for Southern hybridisation was the vls gene cassette. M=length standard.


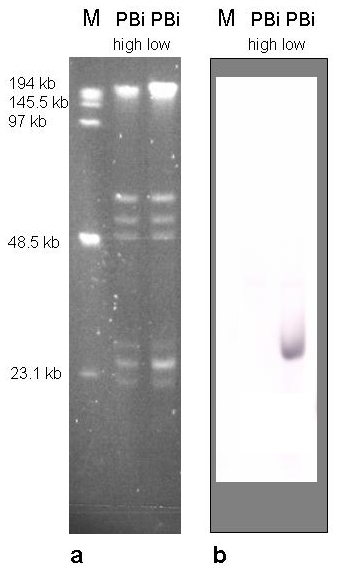

Supplement: Additional File 1 — Analysis of plasmid relationships and losses. The additional file contains tuple plot analysis for all plasmids named in the text and a Southern analysis of the B. garinii PBi low and high passage genome using the vls cassette as a probe. [file 1471-2164-7-211-S1.doc]
